# Effect of Functionalized Benzene Derivatives as Potential Hole Scavengers for BiVO_4_ and rGO-BiVO_4_ Photoelectrocatalytic Hydrogen Evolution

**DOI:** 10.3390/molecules27227806

**Published:** 2022-11-12

**Authors:** Tayebeh Sharifi, Marin Kovačić, Monika Belec, Klara Perović, Marin Popović, Gabrijela Radić, Boštjan Žener, Anamarija Pulitika, Marijana Kraljić Roković, Urška Lavrenčič Štangar, Ana Lončarić Božić, Hrvoje Kušić

**Affiliations:** 1 Faculty of Chemical Engineering and Technology, University of Zagreb, Marulićev trg 19, HR-10000 Zagreb, Croatia; 2 Department of Safety and Protection, Karlovac University of Applied Sciences, Trg Josipa Juraja Strossmayera 9, HR-47000 Karlovac, Croatia; 3 Faculty of Chemistry and Chemical Technology, University of Ljubljana, Večna pot 113, SI-1000 Ljubljana, Slovenia

**Keywords:** BiVO_4_, hydrogen production, photoelectrochemical characterization, aromatic sacrificial agents

## Abstract

Sustainable hydrogen production is one of the main challenges today in the transition to a green and sustainable economy. Photocatalytic hydrogen production is one of the most promising technologies, amongst which BiVO_4_-based processes are highly attractive due to their suitable band gap for solar-driven processes. However, the performance of BiVO_4_ alone in this role is often unsatisfactory. Herein we report the improvement of BiVO_4_ performance with reduced graphene oxide (rGO) as a co-catalyst for the photoelectrochemical water splitting (PEC-WS) in the presence of simple functionalized benzene derivatives (SFBDs), i.e., phenol (PH), benzoic acid (BA), salicylic acid (SA), and 5-aminosalicylic acid (5-ASA) as potential photogenerated hole scavengers from contaminated wastewaters. Linear sweep voltammetry and chronoamperometry, along with electrochemical impedance spectroscopy were utilized to elucidate PEC-WS performance under illumination. rGO has remarkably improved the performance of BiVO_4_ in this role by decreasing photogenerated charge recombination. In addition, 5-ASA greatly improved current densities. After 120 min under LED illumination, 0.53 μmol of H_2_ was produced. The type and concentration of SFBDs can have significant and at times opposite effects on the PEC-WS performance of both BiVO_4_ and rGO-BiVO_4_.

## 1. Introduction

Sustainable energy production and water contamination prevention are one of the key challenges of today. Thus, solving both simultaneously with a single process would be highly advantageous. An attractive process for the accomplishment of both goals is photoelectrocatalysis, thus far it has been investigated for the abatement of organic pollutants and hydrogen and/or oxygen production by water splitting separately [1,2,3,4,5]. In relation to photocatalysis, photoelectrocatalysis suppresses the recombination of photogenerated electron/hole pairs (*e*^−^/*h*^+^) by an external voltage bias, facilitating *e*^−^/*h*^+^ separation and in addition driving the reactions of interest. However, even the external voltage bias does not alleviate the spontaneous recombination of *e*^−^/*h*^+^ completely, which is generally responsible for relatively low quantum yields. In order to suppress charge recombination, and in turn improve both quantum yields and kinetic rates of the reactions of interest, sacrificial agents are commonly utilized as electron acceptors/donors [6,7]. Thus, in the case of photocatalytic water splitting (PC-WS) and subsequent H_2_ generation, using water alone leads to low process efficiency due to a complex multistep reaction involving a four- or two-electron transfer depending on the type of photocatalyst [8]. In addition, water splitting is counteracted by rapid *e^−^/h^+^* recombination and backward oxidation of H_2_ with O_2_ producing water again, and no net gain of H_2_ [7]. However, *h*^+^ scavengers can remarkably improve WS and consequent H_2_ generation by reducing the rate of *e^−^/h^+^* recombination, suppressing the impeding reaction [7]. On the other hand, competing reduction reactions of the oxidation products of the sacrificial agents should be taken into account, which can consequently decrease the H_2_ yield. Previous studies have achieved good results for H_2_ production by PC-WS or PEC-WS using aliphatic alcohols and polyols as *h*^+^ scavengers [9,10]. The idea of using organic pollutants as sacrificial agents, more specifically as *h*^+^ scavengers, has gained increasing attention as an environmentally friendly and potentially ecologically beneficial process [11,12,13,14]. However, to the best of the knowledge of the authors, aromatic pollutants in the role of potential *h*^+^ scavengers for photocatalytic H_2_ production are yet to be reported. This would yield a twofold benefit, simultaneous degradation of harmful organic pollutants in the aquatic environment with simultaneous production of renewable H_2_. Recently, the use of BiVO_4_-based materials as narrow bandgap semiconductors for solar energy harvesting has gained attention in various applications [15,16,17]. We have previously reported an iso-type BiVO_4_ homojunction that exhibits enhanced photocatalytic activity compared to other BiVO_4_ analogs [18,19].

In this work, the photoelectrochemical properties of BiVO_4_ and the influence of an underlying layer of reduced graphene oxide (rGO), as a sink for photogenerated *e*^−^, in a two-layer composite were investigated to evaluate the activities for simultaneous degradation of aromatic pollutants and their potential for the production of H_2_ under visible light irradiation. Phenol (PH), benzoic acid (BA), salicylic acid (SA), and 5-aminosalicylic acid (5-ASA) were selected, as they are common intermediates in the fine chemical and pharmaceutical industries and are known to be harmful to the aquatic environment. The objective was to determine the role of the substituent group in the aromatic ring towards effective *h*^+^ scavenging and improved WS efficiency, indicating improved generation of H_2_, while achieving simultaneous remediation of contaminated water. Therefore, the inclusion of rGO in the composite and the presence of SFBDs in water are hypothesized to yield simultaneous *e^−^/h^+^* scavenging, thereby significantly improving the photoelectrocatalytic activity of BiVO_4_. Photoelectrochemical (PEC) characterization of BiVO_4_ and rGO-BiVO_4_ in conjunction with density functional theory (DFT) calculations were utilized to elucidate underlying mechanisms for photo-electrochemical WS. This study shows how PEC characterization can be utilized to identify the viability of organic contaminants for improving PEC-WS.

## 2. Results and Discussion

### 2.1. DFT Study of SFBDs and Their Interactions with rGO

To determine the potential of the aforementioned SFBDs as hole scavengers in PEC-WS application, the HOMO and LUMO levels of the SFBDs and their relevant aqueous species, namely benzoate (BA^−^) and salicylate (SA^−^) as conjugate bases of their respective acids and the zwitterionic form of 5-ASA, as shown in Figure S1 (supplementary information), were calculated by DFT. The reactivity descriptors calculated according to Equations (2)–(5) can be found in Table 1. The electrophilicity index reveals that all of the SFBDs are electrophilic, in the following descending order: BA > SA > 5-ASA > PH. Moreover, BA and SA have relatively high ionization energies, i.e., -*E*_HOMO_ (Figure S1), hence they are to be expected as the most resistant to oxidation, even more so in the case of BA, which is less soft than SA. In addition, PH has the largest calculated HOMO-LUMO gap amongst the studied SFBDs, thus it will be less susceptible to oxidation as well. Although BA and SA appear to be poor candidates among the selected SFBDs, their conjugate bases are significantly less electrophilic, have markedly lower ionization energies, and are somewhat softer than their non-dissociated counterparts. In other words, they may be more susceptible to oxidation by *h*^+^. In addition, the frontier HOMO of BA^−^ and SA^−^ are localized on the carboxyl group, facilitating adsorption onto BiVO_4_ and consequent oxidation by surface-trapped *h*^+^. 5-ASA is quite electrophilic, as the electron-withdrawing effect of the carboxyl group is only moderately attenuated despite the presence of two electron-donating groups.

However, 5-ASA is the softest among the SFBDs studied and has the lowest ionization potential, making it the most susceptible to polarization and oxidation per se. Since 5-ASA is predominantly present in zwitterionic form in solution under the investigated conditions, it is less soft than its parent, albeit the zwitterion is still softer than other SFBDs. On the other hand, akin to BA^−^ and SA^−^, the frontier HOMO orbital of the zwitterion is favorably situated on the carboxyl group as well. In the case of 5-ASA, the HOMO is most prominent in the aromatic ring and extends to the carboxyl group, although the density is lower than in the case of the zwitterion. PH is the least electrophilic of the neutral SFBDs species, as expected by the contribution of the sole electron-donating hydroxyl group. The ionization energy of PH is lower than that of BA and SA, but it is the least soft among the SFBDs, although only slightly compared with BA. Thus, the most suitable *h*^+^ scavengers seem to be the conjugate bases of aromatic carboxylic acids, even more so than aromatic alcohols. However, the extent of dissociation of aromatic carboxylic acids in solution might play a key role, since the equilibrium concentration of the favorable species towards *h*^+^ oxidation will be relatively low.

**Figure 1:**
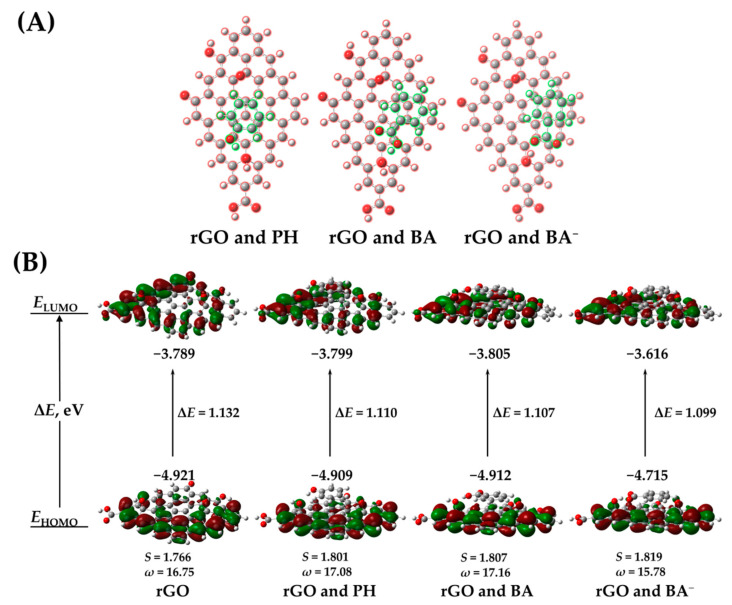
Stacking of PH, BA and BA^−^, respectively, onto rGO (**A**), isosurface plots of HOMO and LUMO frontier orbitals of rGO and dimers: rGO and PH, rGO and BA, rGO and BA^−^ (**B**).

The adsorption of SFBDs on rGO and the resulting electronic interactions may have a profound effect on the charge transfer properties of the rGO-BiVO_4_ composite. For the study of stacking interactions, PH, BA, and BA^−^ were chosen, as they represent SFBDs with significant differences in descriptors and potentially opposing scenarios. The stacking interactions of SFBDs with rGO are also affected by the electrostatic interactions of functional groups on rGO, presumably predominantly with hydroxyl groups. Despite the disruption of the graphene-associated *sp*^2^ framework in rGO due to the presence of oxygen moieties and potential antagonistic electrostatic interactions, some intact *sp*^2^ hybridized domains facilitate the stacking of SFBDs as can be seen in Figure 1A. PH prefers an A-B stacking configuration whereas BA and BA^−^ prefer the so-called bridge stacking configuration [20]. Stacking interactions affect the ionization energies of rGO, as shown in Figure 1B. The effects of PH and BA on the frontier orbitals are much less pronounced than those of BA^−^, significantly reducing the ionization potential. PH and BA increase the softness of the complex with rGO, promoting polarizability and electrophilicity. BA^−^ somewhat decreased the electrophilicity of the dimer, although the BA^−^-rGO complex is still very electrophilic, which may have a certain impact on the photogenerated electron transfer characteristics. On the other hand, BA^−^ may promote electron mobility within rGO by increasing its softness and thereby indirectly decreasing recombination rates of photogenerated charges, facilitating photoelectrocatalytic activity towards WS and pollutant degradation.

### 2.2. Characterization of the Photoelectrode Morphology and Microstructural Characterization of BiVO_4_ and rGO Materials

**Figure 2:**
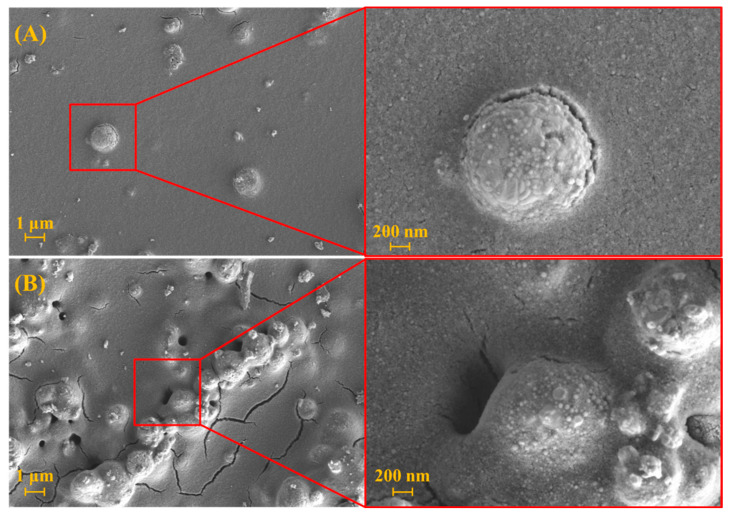
SEM images (top view) of BiVO_4_ (A) and rGO-BiVO_4_ (B) photoelectrodes.

**Figure 3:**
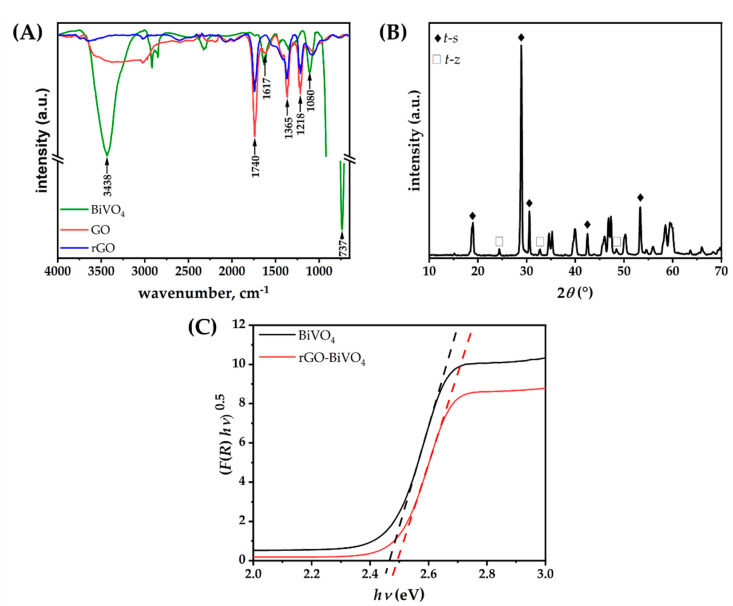
(A) FTIR, (B) XRD of BiVO_4_, and (C) Tauc plots using Kubelka–Munk transformation for BiVO_4_ and rGO-BiVO_4_ photoelectrode.

The morphology of the BiVO_4_ and rGO-BiVO_4_ photoelectrodes are shown in Figure 2. As can be seen, the surface of the BiVO_4_ photoelectrode is less rough than that of the rGO-BiVO_4_ photoelectrode with fewer surface defects. BiVO_4_ agglomerates are more prominent on the surface of the rGO-BiVO_4_ photoelectrode. Moreover, the layer porosity in the form of cracks and pinholes on the surface of rGO-BiVO_4_ was presumably caused due to desorption of the adsorbed solvent on the rGO layer underneath during post-coating heat treatment. The linear heat expansion coefficient of rGO is of a similar value to borosilicate glass [21], hence cracking should not occur due to dissimilar expansion under heating. The observed surface defects may act as channels for the diffusion and adsorption of the SFBDs on the rGO layer underneath. The FTIR spectra of the prepared BiVO_4_ and rGO are shown in Figure 3A.

The characteristic IR band of BiVO_4_ was observed at 700 cm^−1^ to 900 cm^−1^, centered at 737 cm^−1^ [22]. The FTIR spectrum of rGO indicated that incomplete reduction in GO has occurred, as absorption maxima corresponding to oxygen moieties identified in GO have been detected in rGO as well, albeit with lower absorption intensities. Carbonyl stretching originating from carboxyl, aldehyde, or ketone groups at 1740 cm^−1^, C–O stretching vibrations of epoxy and alkoxy groups at 1218 cm^−1^ and 1080 cm^−1^, respectively, and C-OH vibrations at 1365 cm^−1^ indicate the presence of oxygen-functionalized moieties. The C=C band identified at 1617 cm^−1^ can be attributed to the bending of the C=C bonds in the aromatic moieties within rGO [23,24]. The strong O–H stretching vibration band in the range of 3000 cm^−1^ to cm^−1^, corresponding to adsorbed water, is not as pronounced for rGO as for GO and BiVO_4_ [25]. To further support these findings, thermogravimetric (TGA) analysis of both GO and rGO was performed (Figure S2 (supplementary information)). The weight loss recorded for rGO in both N_2_ and air up to 150 °C is minuscule in comparison to GO, thus confirming that rGO contains substantially less adsorbed water. In addition, the mass loss from 150 °C up to 350 °C in N_2_ for GO is nearly 51.9%, whereas rGO lost 43.1% of its initial mass up to that point. The difference indicates the extent of reduction in rGO, i.e., the relatively lower concentration of oxygen moieties in rGO. The carbonaceous residue at 800 °C in N_2_ for GO equals 21.2% of the initial mass, whereby for rGO it equals 47.9%, which correlates well to the relative ratio of the FTIR absorption peak heights for carbonyl groups observed in Figure 3A. Upon oxidation in the air as per Figure S2B, the residual mass for both GO and rGO is nil, revealing that the post-synthesis purification of both samples was carried out diligently. Further structural characterization of the prepared materials was performed by XRD. As can be seen in diffractograms of the prepared materials (Figure 3B), two distinct phases were found for BiVO_4_: a scheelite-type structure crystallizing in the tetragonal system (*t*-*s*), which is in good agreement with published diffraction data (ICDD 01–074-4892), and zircon type structure crystallizing in the tetragonal system (*t*-*z*), matching (ICDD 00–014-0133) [18,26]. The strongest diffraction peaks at 2*θ* = 18.6°, 19°, 28.8°, 28.9°, 30.5°, 34.5°, 35.2°, 39.8°, 42.4°, and 53.3° correspond to planes (101), (011), (013), (112), (004), (200), (020), (211), (015), and (116) of the *t*-*s* phase, crystallizing in the space group *I2*/*b*. Moreover, the peaks at 2*θ* = 24.4°, 32.7°, and 48.5° correspond to (200), (112), and (312) crystal planes of the *t*-*z* phase, which crystallizes in the space group *I41*/*amd*. The *t*-*s* phase is the predominant phase, whereas the *t*-*z* phase is present as a minor constituent, confirming the synthesis of an isotype homojunction BiVO_4_ [18]. The diffractograms of GO and rGO are provided in Figure S3 (supplementary information). Distinct diffraction maxima corresponding to the (001) plane of GO at 2*θ* = 13.5° and (002) plane of rGO at 2*θ* = 23.8° can be seen, indicating a smaller lower inter-layer sheet distance within rGO due to the reduction in oxygen-containing moieties [27,28]. Thus XRD data confirmed the findings of GO reduction drawn from FTIR and TGA analysis. The Tauc plots conducted on the diffuse reflectance spectra for BiVO_4_ and rGO-BiVO_4_ photoelectrodes are shown in Figure 3C. As can be seen, the calculated band gap values (*E*_g_) are similar; 2.47 eV and 2.50 eV were obtained for BiVO_4_ and rGO-BiVO_4_, respectively.

### 2.3. Photoelectrochemical Characterization

**Figure 4:**
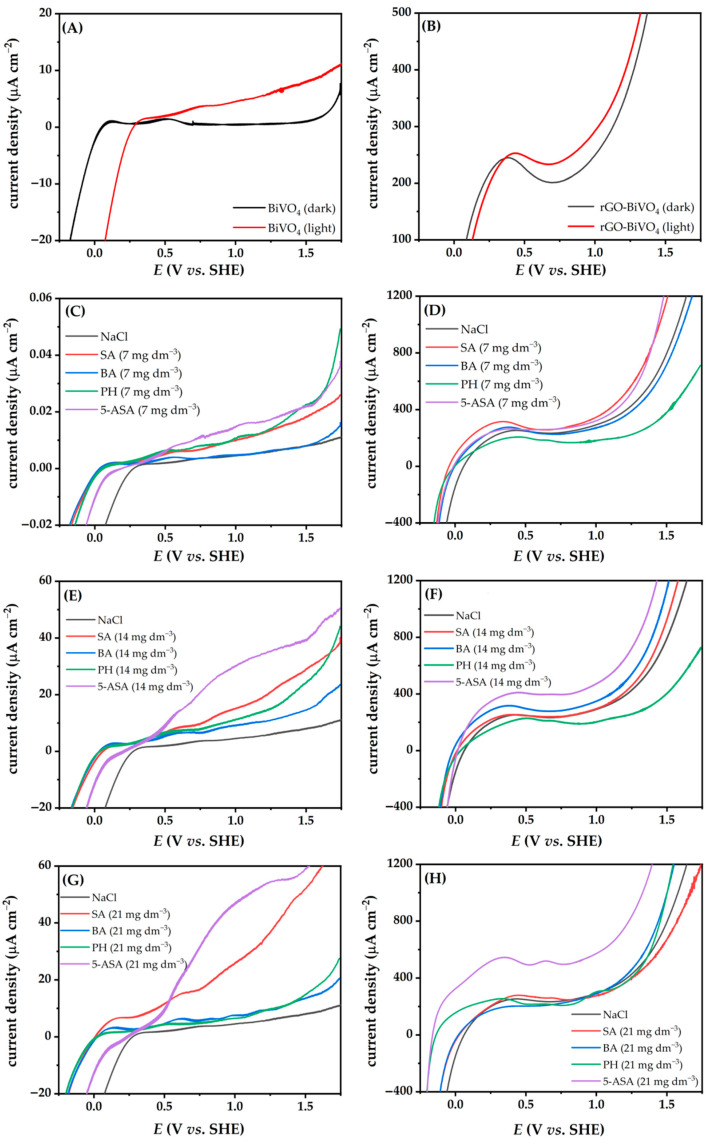
Linear sweep voltammetry (LSV) of BiVO_4_ (**A**) and rGO-BiVO_4_ (**B**) photoelectrodes in NaCl solution (0.1 M) in the dark and under illumination, LSV of BiVO_4_ in NaCl solution containing SFBDs (**C**,**E**,**G**) and LSV of rGO-BiVO_4_ (**D**,**F**,**H**) under illumination.

In order to achieve the required high current densities for good PEC performance, two parameters need to be met: (i) effective *e^−^/h^+^* separation and (ii) good charge carrier mobility. The polarization curves (from −0.26 to 1.74 V vs. SHE) for BiVO_4_ (three layers) and rGO-BiVO_4_ (one layer rGO and two layers BiVO_4_) in NaCl (0.1 M) containing different concentrations of SFBDs are shown in Figure 4 and Figure S3 (supplementary information). As shown in Figure 4A, the current density of BiVO_4_ is near zero in the dark in NaCl solution (Figure 4A). Upon illumination, band bending of the BiVO_4_ photoelectrode occurs, thus the photocurrent increases with increasing potential during the linear voltage sweep. However, a high current density was obtained for the rGO-BiVO_4_ photoelectrode in the dark (Figure 4B), since the rGO layer can act as a conductive layer due to its high work function and the presence of delocalized *sp*^2^ networks [29,30,31,32]. The peak observed at 0.1 V in Figure 4B vs. SHE in the dark is a result of the pseudocapacitive behavior of oxygen-containing functional groups of rGO [33,34]. The significant current increase observed for the rGO-BiVO_4_ photoelectrode in the NaCl solution at potentials greater than 0.8 V is a compounded effect of photocurrent increase, as well as a contribution of the redox reaction of rGO, i.e., oxidation of rGO. In the presence of the SFBDs in the NaCl solution, the current density of BiVO_4_ was near zero in the dark, except in the case of 5-ASA, which can be attributed to electrooxidation of 5-ASA (Figure S3 (supplementary information)). DFT calculations have indicated that the zwitterionic form of 5-ASA has the lowest ionization potential, hence such a finding is unsurprising. Under illumination, the current density or rGO-BiVO_4_ increased due to the generation of *e^−^/h^+^* pairs, even more so than in the case of the BiVO_4_ photoelectrode, indicating good photoactivity and the beneficial effect of rGO on the composite photoelectrode. The current densities of the photoelectrodes increased more than that of the pure electrolyte after introducing the lowest concentration of the SFBDs upon illumination (Figure S3 (supplementary information)). A lower photocurrent density recorded in the pure electrolyte indicates a higher recombination rate, thereby prohibiting electrons from reaching the counter electrode through the external circuit. Oxidation of SFBDs by *h*^+^, acting as sacrificial *e*^-^ donors on the surface of the photoelectrodes, has improved charge separation. The influence of the initial concentration of the SFBDs (7, 14, and 28 mg dm^−3^) on PEC activities was investigated (Figure 4 and Figure S4 (supplementary information)). In the case of BiVO_4_, the current densities increased with the introduction of PH, 5-ASA, and SA. However, at a concentration higher than 14 mg dm^−3^, the current density in the presence of PH decreased significantly. The observed effect can be attributed to decreasing electrophilicity of rGO upon PH multi-layer stacking [35] at higher concentrations, resulting in unfavorable electrostatic interactions. Furthermore, the observed phenomena can be due to the presumably larger surface area of rGO-BiVO_4_ photoelectrode (namely, rGO has three times higher surface area than BiVO_4_ [18,36] and the contributing effect of adsorption of the SFBD in higher concentration since the surface of the photoelectrocatalyst reaches saturation thereby decreasing the PEC performance. BA did not significantly affect the LSV curves of BiVO_4_ and the effect on current density is not as prominent as in the case of PH with changing concentration. Hence, greater electrophilicity of undissociated BA and its highest ionization energy may impede oxidation by *h*^+^, whereas the equilibrium concentration of BA^−^ may be nearly constant despite increasing BA concentrations. On the other hand, SA is significantly softer and has a lower ionization potential, which positively affects oxidation via *h*^+^. In the case of SA and 5-ASA at higher concentrations (28 mg dm^−3^), both have demonstrated higher current densities indicating favorable reactions toward H_2_ evolution. In the case of rGO-BiVO_4_ photoelectrodes, different effects on the PEC properties were observed in relation to BiVO_4_. The inverse effect of the concentration of PH on the photocurrent was observed; a lower concentration of PH achieved higher current density. This may be ascribed to the relative stability of PH towards oxidation as predicted by quantum chemical descriptors, as discussed above. Thus, the adsorption of PH onto the photoelectrode blocks active sites. As mentioned earlier, the current density of photoelectrodes depends not only on the inherent *h*^+^ scavenging properties of the SFBDs but also on the concentration thereof. These trends can be explained by their adsorption affinity and their respective oxidation mechanisms. Adsorption is well known to decrease recombination by promoting the consumption of photogenerated charges via redox reactions with the adsorbate. However, a further increase in the concentration of the adsorbate on the surface of photoelectrocatalyst above the optimum can lead to a blockage of active sites and a consequent decrease in activity.

Moreover, if the oxidation proceeds rapidly, electron transfer from the oxidized product into the conduction band of the photoelectrode leads to a more pronounced current increase, i.e., the so-called current doubling effect [37]. Further oxidation of the oxidized species on the surface of the photoelectrode is possible, thereby injecting more electrons into the conduction band. The results revealed that SA and 5-ASA result in higher current densities than those achieved with PH and BA as *h*^+^ scavengers, thus SA and 5-ASA are better sacrificial agents for H_2_ production as demonstrated by PEC. The effect is even more pronounced at higher concentrations (Figure 4F,H). These results support the role of the different substituent groups in the aromatic ring as suggested by the intuition based on organic chemistry principles, which implies electron withdrawal from the aromatic π-system aromatic system, in turn reducing the electron density. On the other hand, amino and hydroxyl substituents of 5-ASA enrich the π-system electron density, thereby making these SFBDs more suitable for *h*^+^ scavenging. A two-fold effect is achieved, i.e., innate photoelectrochemical production of H_2_ is enhanced due to prolonged half-lives of the photogenerated *e*^−^. These *e*^−^ are then more likely to migrate to the Pt cathode and perform the reaction of interest. Second, such SFBD is susceptible to photoelectrocatalytic oxidation, thus leading to *e*^−^ injection into the photoelectrode. 

**Figure 5:**
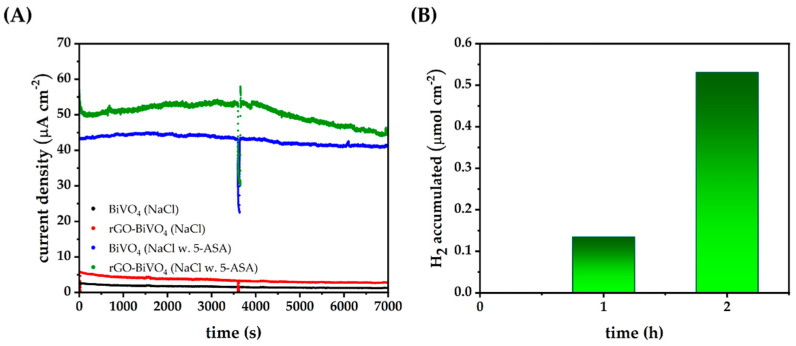
(**A**) Chronoamperometry at 0.6 V bias potential under constant illumination of BiVO_4_ and rGO-BiVO_4_ electrodes in 0.1 M NaCl and 0.1 M NaCl with *γ*(5-ASA) = 14 mg dm^−3^, (**B**) accumulated H_2_ by rGO-BiVO_4_ in chronoamperometric experiments in 0.1 M NaCl with *γ*(5-ASA) = 14 mg dm^−3^.

Further studies encompassed chronoamperometry of BiVO_4_ and rGO-BiVO_4_ electrodes under constant illumination in the presence of 5-ASA at a concentration of 14 mg dm^−3^ in order to quantify the evolved H_2_. At first, freshly prepared photoelectrodes displayed a high initial current, which under illumination decreased quickly to relatively stable values even in consequent cycles. In Figure 5A chronoamperograms of previously used BiVO_4_ and rGO-BiVO_4_ photoelectrodes immersed in 0.1 M NaCl electrolyte and the electrolyte containing 5-ASA are shown. The photoelectrodes were rinsed with deionized water and dried in between cycles. As can be seen, rGO enhances the photocurrent response of BiVO_4_ in 0.1 M NaCl and throughout the experiment the photoelectrode remains reasonably stable, indicating that rGO is not degraded, at least not to a large extent. The enhanced current density of rGO-BiVO_4_ in relation to BiVO_4_ in NaCl is a direct result of the inhibition of *e^−^/h^+^* recombination. Upon the addition of 5-ASA, dramatic increases in photocurrents are observed for both BiVO_4_ and rGO-BiVO_4_, as supported by LSV measurements shown above. However, without 5-ASA the quantity of evolved hydrogen in both cases is below the instrumental detection limit. In addition, the quantity of hydrogen evolved by BiVO_4_ alone is minuscule and below the instrumental quantification limit. The observed phenomenon can be ascribed to fast *e^−^/h^+^* recombination in BiVO_4_ alone [38].

After 120 min in the case of rGO-BiVO_4_, 0.53 μmol of hydrogen was accumulated, as shown in Figure 5B. A decrease in the current densities of the rGO-BiVO_4_, and to some extent in BiVO_4_, after 1 h (3600 s) of illumination can be ascribed to the accumulation of 5-ASA degradation byproducts in the vicinity of the photoelectrode and to their adsorption to the photoelectrode surface. Thus, the previously discussed current doubling effect diminishes as oxidizable species are depleted from the solution. However, the apparent rate of hydrogen production is not negatively affected, in fact, the apparent rate has increased as shown in Figure 5B.

**Figure 6:**
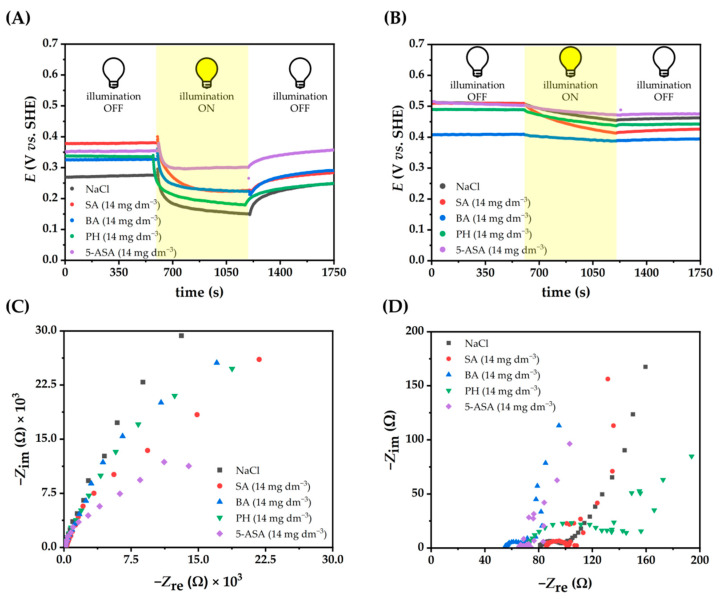
OCP of BiVO_4_ (**A**) and rGO-BiVO_4_ (**B**) photoelectrodes, EIS of BiVO_4_ (**C**) and rGO-BiVO_4_ (**D**) under illumination in NaCl (0.1 M)-containing SFBDs.

In order to further investigate the kinetics of *e^−^/h^+^* recombination electrochemically, open circuit potentials (OCPs) of BiVO_4_ and rGO-BiVO_4_ photoelectrodes were measured in 0.1 M NaCl solution containing different concentrations of PH, BA, SA, 5-ASA (Figure 6 and Figure S5 (supplementary information)).

As can be seen, a negative shift in the potential was observed for all electrodes upon illumination, indicating that the photogenerated *h*^+^ react rapidly with the electrolyte on the surface of the electrode, whereas the photogenerated *e*^-^ lower the potential of the photoelectrodes. In the case of the BiVO_4_ photoelectrode, the OCP increased in the dark in NaCl-containing SFBDs. However, in the case of rGO-BiVO_4_, there was no similar trend and the OCP value changed for each SFBD at different concentrations. Moreover, the photopotential profiles of rGO-BiVO_4_ photoelectrodes showed slow kinetics of charge transfer across the photoelectrodes, resulting in a stable OCP value observed under 10 min light illumination. The same behavior has been reported for other materials based on rGO, or materials with multiple surface states [39], in which the charge carriers are trapped. It is worth noting that the rGO-BiVO_4_ photoelectrodes also exhibited supercapacitor-like behavior, manifested by a slow decay part of the OCP value after the illumination was stopped, which can be attributed to the presence of an rGO layer in the photoelectrode. Thereby, rGO is an effective co-catalyst, promoting the activity of BiVO_4_ by acting as a sink for photogenerated *e*^-^ and hindering recombination.

The charge transfer resistances were determined by electrochemical impedance spectroscopy (EIS) in NaCl solutions with dissolved SFBDs (Figure 6C,D and Figure S5 (supplementary information)). Comparison of the Nyquist plots in the dark and under illumination in the presence of SFBDs revealed that the arc radii of the Nyquist semi-circles have decreased under illumination in comparison to measurements performed in the dark (Figure S5 (supplementary information)). Such results indicate enhanced electron transfer to the external circuit that suppresses *e^−^/h^+^* pair recombination, which is in good agreement with LSV results. Moreover, the arc radii of the Nyquist plot under illumination were affected by the concentration of SFBDs. For the BiVO_4_ photoelectrodes, the lowest concentration of PH, the middle concentration of BA, and the highest concentration of SA and 5-ASA (Figure S5 (supplementary information)) resulted in the smallest arc radii. In the case of rGO-BiVO_4_ photoelectrodes, the highest concentration of PH, middle concentration of BA, and the lowest concentration of SA and 5-ASA resulted in smaller arc radii (Figure S5 (supplementary information)). Resistance (*R*_s_) and charge transfer resistances (*R*_ct_) are given in Table 2. The SFBDs resulting in LSV currents larger than for NaCl alone had unsurprisingly lower charge transfer resistances. The greater charge transfer resistance observed for PH in the case of rGO-BiVO_4_ photoelectrode is indicative of unfavorable photoelectrocatalytic oxidation of PH on the surface of the photoelectrode.

**Figure 7:**
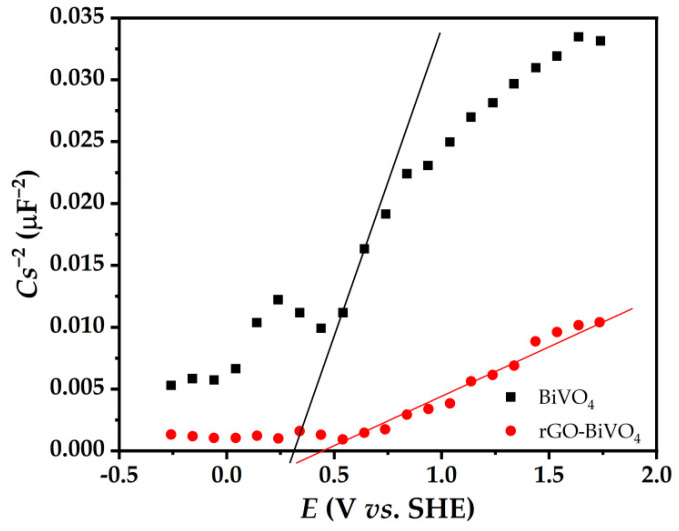
Mott–Schottky plots of BiVO_4_ and rGO-BiVO_4_ photoelectrodes.

Mott–Schottky analysis was also performed and the result is shown in Figure 7. The values of the flat band potential (*E*_fb_) and electron donor density (*N*) can be extracted from Equation (1) for BiVO_4_ and rGO-BiVO_4_ photoelectrodes by measuring the capacitance of the space charge region of photoelectrode/electrolyte junction (*C*) for different applied photoelectrode potentials in the dark and in NaCl (0.1 M) solution:

(1) $$\frac{1}{C^{2}}=\frac{2}{\left(e\varepsilon_{0}\varepsilon NA^{2}\right)}\left(E_{\mathrm{app}}-E_{\mathrm{fb}}-\frac{kT}{q}\right)$$

The *E*_fb_ was determined by extrapolation on the x-axis. In addition, the slope is inversely proportional to the electron donor density (*N*), where *e*, *ε*_0_, *ε*, *A*, *E*_app_, and *kT*/*q* are the electron charge, the dielectric permittivity of vacuum, the dielectric constant of the semiconductor, the area of the sample exposed to the electrolyte, the applied potential, and the temperature-dependent term, respectively.

As shown in Figure 7, the *E*_fb_ of rGO-BiVO_4_ is 0.44 V vs. SHE, which is more positive than that of BiVO_4_ (0.29 V vs. SHE). The Mott–Schottky plot of rGO-BiVO_4_ also shows a much lower slope than that of BiVO_4_, which is due to the higher specific surface area of the rGO-BiVO4 photoelectrode compared to BiVO_4_. The high electrical conductivity of the rGO layer makes it an excellent acceptor for photogenerated *e^−^*, which enhances the PEC activity through effective and rapid photogenerated *e^−^/h^+^* separation.

## 3. Materials and Methods

### 3.1. Synthesis of BiVO_4_

Bismuth(III) nitrate pentahydrate (Bi(NO_3_)_3_×5H_2_O, VWR Chemicals, Paris, France), ammonium metavanadate (NH_4_VO_3_, Acros Organics, Geel, Belgium), ethylenediaminetetraacetic acid disodium salt dihydrate (EDTA-2Na, T.T.T., Sveta Nedjelja, Croatia), and nitric acid (HNO_3_, Lach-ner, Czech Republic) were used for BiVO_4_ synthesis. Ultrapure water from a Milli-Q system (Direct-Q3 UV, Merck Millipore, Burlington, MA, USA) was used to prepare all aqueous solutions. BiVO_4_ was synthesized using a co-precipitation method [18]. Briefly, 0.2 M solutions of Bi(NO_3_)_3_×5H_2_O and NH_4_VO_3_ were prepared in HNO_3_ (1.5 M) and hot water (80 °C), respectively. A total of 0.382 g of EDTA was dissolved in 50 mL of Bi(NO_3_)_3_ solution. The precursor solutions were then mixed and stirred for one week at room temperature in a dark environment. BiVO_4_ products were isolated using a centrifuge (EBA 21, Hettich Zentrifugen, Zürich, Switzerland) at a relative centrifugal force of 1370*g* × for 3 min, washed three times with ultrapure water, dried at 80 °C in a laboratory oven (UN-55, Memmert, Schwabach, Germany), and finally calcined at 450 °C for 2 h in a laboratory furnace (LP-08, Instrumentaria, Zagreb, Croatia).

### 3.2. Synthesis of GO and rGO

Graphene oxide (GO) was prepared by the Hummers procedure [40]. Briefly, 3.0 g of graphite powder (Merck, Darmstadt, Germany) and 1.5 g sodium nitrate (NaNO_3_, Merck) were added to 69 mL of ice-cold concentrated sulfuric acid (H_2_SO_4_, 96%, Kemika, Zagreb, Croatia). A total of 9.0 g of potassium permanganate (KMnO_4_, Merck) was then gradually added to the solution, with continuous stirring and at a temperature below 20 °C for 20 min. The flask was then placed in a water bath at 35 °C and stirred for another 30 min. Then 120 mL of deionized water was slowly added to the solution and stirred at 98 °C for 30 min. The flask was then cooled down to room temperature and the mixture was poured into 420 mL deionized water with vigorous stirring. This was followed by the addition of 20 mL of 30% H_2_O_2_ in order to remove the excess oxidant and quench the reaction. The obtained mixture was carefully washed with 5% hydrochloric acid (HCl, Gram-mol) until the pH value approached 7.0 and centrifuged to remove the residual precipitated salts. Finally, the obtained GO product was purified by dialysis to further remove inorganic impurities and consequent centrifugation to remove any aggregates. rGO was obtained from GO by a hydrothermal reduction procedure. A homogeneous GO suspension in EtOH (1 mg mL^−1^) was prepared by ultrasonic homogenization using an EW-08848-15 ultrasonic bath (Cole Parmer, Vernon Hills, IL, USA). A total of 19 mL of the GO suspension was transferred to a Teflon-lined stainless autoclave, then 1 mL of acetic acid and 0.2 g of thioacetamide (TAA, Merck) were added to the suspension, the autoclave was then sealed and heated to 180 °C for 12 h. The product was isolated by centrifugation, washed, and dried at 100 °C.

### 3.3. Photoelectrode Preparation

2.2 mm thick fluorine-doped tin oxide glass (FTO, Sigma-Aldrich, St. Louis, MO, USA) with a resistivity of 7 Ω/sq was used as a working electrode, with a surface of 1 cm^2^ in photoelectrochemical (PEC) tests. Acetone ((CH_3_)_2_CO, 99.9%, Gram-mol) and ethanol (CH_3_CH_2_OH, EtOH, abs., Sigma-Aldrich) were used to clean the surface of FTO glass. Titanium tetraisopropoxide (Ti[OCH(CH_3_)_2_]_4_, TTIP, 97%, Sigma-Aldrich), tetraethyl orthosilicate (Si(OC_2_H_5_)_4_, TEOS, 99% GC grade, Sigma-Aldrich), EtOH, HCl, and Levasil^®^ 200/30 (colloidal SiO_2_, Obermeier, Bad Berleburg, Germany) were used to prepare a silica-titania immobilization sol for spin coating of BiVO_4_ onto the surface of FTO glass [41]. Firstly, the FTO glass was separately sonicated for 10 min in acetone, EtOH, and Milli Q water and then dried at room temperature. Then 0.1 g of BiVO_4_ was dispersed in 600 μL of the titania/silica binder [19,41]. In order to obtain the BiVO_4_ photoelectrode, 100 μL of the aforementioned dispersion was deposited using a spin coating procedure at 1500 rpm (KW-4A spin coater, Chemat Technology, Northridge, CA, USA). Three layers of BiVO_4_ were deposited by spin coating and dried at 200 °C for 2 h in an UN-55 laboratory oven (Memmert, Germany). In the case of the rGO-BiVO_4_ photoelectrode, first 0.3 g of rGO was dispersed in 10 mL of EtOH and 15 μL of the rGO/EtOH dispersion was dropped onto the surface of FTO glass and dried at room temperature. Then two consecutive layers of BiVO_4_ were coated by spin coating like the above-mentioned procedure.

### 3.4. Photocatalyst Characterization

Morphological studies were conducted by an Ultra Plus scanning electron microscope (SEM) (Zeiss, Germany). Structural characterization of BiVO_4_, GO and rGO was performed using X-ray diffraction (XRD) on a Miniflex 600 equipped with D/teX Ultra 2 silicon strip detector (Rigaku, Tokyo, Japan). Fourier transform infrared (FTIR) spectra were recorded on a Spectrum One spectrometer (Perkin Elmer, Waltham, MA, USA) in the range of 4000 cm^−1^ to 650 cm^−1^ using attenuated transmission reflectance technique. The diffuse reflectance measurements were taken on FTO-coated photoelectrodes using a UV-VIS spectrometer 2600i equipped with an ISR-2600Plus integrating sphere (Shimadzu, Kyoto, Japan).

### 3.5. Photoelectrochemical (PEC) Measurements

PEC measurements of the prepared photoelectrodes were performed using an SP-150 potentiostat/galvanostat (Biologic, Seyssinet-Pariset, France). Experiments were performed in a typical three-electrode setup consisting of the as-prepared BiVO_4_ or rGO-BiVO_4_-coated FTO glass as the working electrode, a platinum (Pt) counter-electrode, and a saturated calomel reference electrode (SCE) at natural pH of the SFBD solutions. All of the potentials reported were converted to standard hydrogen electrode (SHE) potential. A LED with a peak wavelength of ~550 nm was used as the irradiation source [19]. Linear sweep voltammetry (LSV) with a scan rate of 20 mV s^−1^ and open circuit potential was measured in the dark and under illumination. Chronoamperometry was performed at a 0.6 V bias potential. The electrochemical impedance spectra (EIS) of the samples were measured at frequencies ranging from 100 kHz to 100 mHz at 0 V vs. SCE and an AC voltage amplitude of ±5 mV in the dark and under light illumination. The Mott–Schottky measurements were also carried out to determine the position of the flat band potential as well as the donor concentration of BiVO_4_ and rGO-BiVO_4_ photoelectrodes at different frequencies. All above-mentioned tests were performed in a solution of sodium chloride (0.1 M, NaCl, 99%, Sigma-Aldrich) containing three different concentrations (7, 14, and 28 mg dm^−3^) of phenol (PH, 99.5%, Sigma-Aldrich), benzoic acid (BA, 99%, Kemika), salicylic acid (SA, 99%, Kemika), and 5-amino salicylic acid (5-ASA, 99%, Acros Organics). Evolved hydrogen (H_2_) during the photoelectrocatalytic experiments was detected and quantified using an Autosystem XL gas chromatograph (Perkin Elmer, USA), equipped with a thermal conductivity detector (TCD). A total of 300 μL of the gas from the headspace above the Pt counter-electrode was sampled via syringe and injected in a splitless mode at an injector temperature of 120 °C. Chromatographic separation of the gases was achieved on a ShinCarbon ST 100/120 (Restek, Bellefonte, PA, USA) 2 m, 1 mm I.D., packed column using argon as carrier gas at a flow rate of 10 mL min^−1^. A 0.1% *v*/*v* mixture of H_2_/Ar (Uljanik Tehnički Plinovi, Pula, Croatia) was used for calibration.

### 3.6. Density Functional Theory Calculations

Density functional theory (DFT) calculations were performed to obtain reactivity descriptors of the investigated SFBDs and to elucidate interactions between the SFBDs and rGO. Structural relaxation of the initial structures of SFBDs and their corresponding reactivity descriptor calculations were performed by Gaussian 16 [42] using the B3PW91 functional and 6-311G(d, p) basis set [43,44]. Tight convergence criteria for both the geometry and self-consistent field procedure were used. The effect of the aqueous phase on the structural properties of the SFBDs was included using the polarizable continuum model (PCM) within Gaussian [45]. Frontier orbitals, i.e., the highest occupied molecular orbital (HOMO) and the lowest unoccupied molecular orbital (LUMO) were used to elucidate potential reactivity of SFBDs towards *h*^+^ by calculating reactivity descriptors and qualitative chemical concepts such as electronegativity (*χ*), chemical hardness (*η*), softness (*S*), and electrophilicity (*ω*) indices according to Equations (2)–(5) [46,47]:

(2) $$\chi =\left(\frac{-E_{\mathrm{HOMO}}-E_{\mathrm{LUMO}}}{2}\right)$$

(3) $$\eta =\left(\frac{E_{\mathrm{LUMO}}-E_{\mathrm{HOMO}}}{2}\right)$$

(4) $$S=\frac{1}{\eta }$$

(5) $$\omega =\frac{{\left(\frac{E_{\mathrm{HOMO}}+E_{\mathrm{LUMO}}}{2}\right)}^{2}}{2\eta }$$

Interactions of the selected SFBDs and rGO were studied by optimizing their interacting molecular structures *in vacuo* utilizing the same function as aforementioned calculations, albeit with a 6-31G(d,p) basis set with included Grimme’s D3 empirical dispersion [48] and default geometry convergence criteria in order to reduce computational costs. An oxygen-functionalized structure of rGO was adopted from the paper of Maity et al. [49]. A superfine integration grid was used for numerical integrations during geometry optimization. Single point energy calculations of the optimized structures were performed using the 6-311G (d,p) basis set with included PCM.

## 4. Conclusions

rGO acted as an effective sink for photogenerated *e*^−^ by BiVO_4_ thereby inhibiting recombination, which was further suppressed by selected SFBDs such as BA, SA, and 5-ASA. In addition to *h*^+^ scavenging by the oxidation of the aforementioned SFBDs on the surface of the photoelectrode, DFT calculations have shown that these SFBDs positively interact with rGO, increasing its electrophilicity and further promoting charge transfer. Thus, the highest current densities were achieved in the case of rGO-BiVO_4_ in the presence of 5-ASA and SA. It is worth noting that several orders of magnitude higher current densities were achieved compared to BiVO_4_ alone, significantly enhancing PEC-WS activity for potential H_2_ generation using rGO-BiVO_4_. Depending on the nature of the substituent in the SFBDs and whether BiVO_4_ or rGO-BiVO_4_ were applied, different effects on PEC-WS activity were observed. Molecular reactivity descriptor concepts obtained by DFT calculations, such as electronegativity, chemical hardness, softness, and electrophilicity have proven themselves as valid indicators for potential *h*^+^ scavenging of the selected compounds. The 5-ASA, followed by SA, was the most perspective candidate for improving PEC-WS activity due to favorable softness and small *E*_HOMO_-*E*_LUMO_ gap indicating a low barrier for oxidation. The 5-ASA significantly improved the PEC-WS activity for both BiVO_4_ and rGO-BiVO_4_, as confirmed by increasing photocurrent densities and measured hydrogen. The effect of BA was strongly dependent on the photoelectrocatalyst; in the case of BiVO_4_ the *h*^+^ scavenging activity of BA was poor due to its high electrophilicity and low softness, and consequently, BA is an ineffective sacrificial agent for PEC-WS. PH surprisingly decreased the PEC-WS activity of rGO-BiVO_4_, as it was the most stable towards photo-electrooxidation among the studied SFBDs. In addition, the adsorption of PH on the photoelectrode likely caused an unfavorable environment for photogenerated *e*^-^ trapping from BiVO_4_ due to π-π stacking of PH on rGO. Additionally, it was found that the concentration of the SFBDs had a significant effect on the overall PEC-WS effectiveness, whereby the optimum PEC-WS effectiveness is a function of the concentration of the SFBDs.

## Figures and Tables

**Table 1:** *E*_HOMO_—*E*_LUMO_ energy gaps (Δ*E*_HOMO-LUMO_, eV) and corresponding reactivity descriptors, i.e., electronegativity (*χ*), chemical hardness (*η*), softness (*S*), and electrophilicity (*ω*) indices of the investigated SFBDs.

DFT Descriptor	PH ^1^	BA ^1^	BA^−,1^	SA ^1^	SA^−,1^	5-ASA ^1^	5-ASA ZW ^1^
Δ*E*_HOMO-LUMO_, eV	−5.932	−5.724	−5.157	−5.076	−5.184	−4.077	−4.752
*χ*	3.456	4.599	3.129	4.163	3.238	3.619	3.456
*η*	2.966	2.884	2.612	2.558	2.612	2.068	2.395
*S*	249.5	256.6	284.2	289.5	282.9	360.4	309.3
*ω*	2.011	3.646	1.888	3.401	2.011	3.157	2.495

^1^ PH—phenol, BA—benzoic acid, BA^−^—conjugated base of benzoic acid, SA—salicylic acid, SA^−^ conjugated base of salicylic acid, 5-ASA—5-aminosalicylic acid, 5-ASA ZW—5-aminosalicylic acid zwitterion.

**Table 2:** Charge transfer resistances (*R*_ct_) obtained from EIS spectra of BiVO_4_ and rGO-BiVO_4_ photoelectrodes in NaCl solution (Figure 6). The impedance spectra were analyzed by complex non-linear least squares regression with modulus weighting using ZSimpWin software.

Resistance	NaCl	PH	BA	SA	5-ASA
*R*_s_(BiVO_4_) (Ω)	69.31	75.27	65.11	37.10	50.77
*R*_s_(rGO-BiVO_4_) (Ω)	81.63	85.09	70.67	54.86	64.45
*R*_ct_(BiVO_4_) × 10^−4^ (Ω)	23.60	10.40	9.32	6.14	2.47
*R*_ct_(rGO-BiVO_4_) × 10^−4^ (Ω)	18.23	69.45	15.22	17.97	7.32

## Data Availability

The datasets collected and analyzed within this work are available from the corresponding authors upon written request.

## Supplementary Materials

The following supporting information can be downloaded at: https://www.mdpi.com/article/10.3390/molecules27227806/s1, Figure S1: isosurface plots of HOMO and LUMO frontier orbitals of PH, BA, BA^-^, SA^-^, 5-ASA, and 5-ASA ZW; Figure S2: diffuse reflectance spectra of BiVO_4_ and rGO-BiVO_4_ photoelectrodes; Figure S3: LSV of BiVO_4_ and rGO-BiVO_4_ photoelectrodes in NaCl solution (0.1 M) containing different organic pollutants of PH (A and B), BA (C and D), SA (E and F), and 5-ASA (G and H) in the dark and under light illumination; Figure S4: OCP of BiVO_4_ (A, C, E, G) and rGO-BiVO_4_ (B, D, F, H) photoelectrodes in NaCl solution (0.1 M) with simple functionalized benzene derivatives; Figure S5: EIS of BiVO_4_ (A, C, E, G) and rGO-BiVO_4_ (B, D, F, H) in the dark and under light illumination in NaCl (0.1 M) with simple functionalized benzene derivatives. Figure S6. EIS of BiVO_4_ (A, C, E, G) and rGO-BiVO_4_ (B, D, F, H) in the dark and under light illumination in NaCl (0.1 M) with simple functionalized benzene derivatives, with inlays corresponding to response in NaCl (0.1 M) alone.

## Sample Availability

Samples of the compounds are not available from the authors.
