# Supplementary material for: Effect of Functionalized Benzene Derivatives as Potential Hole Scavengers for BiVO4 and rGO-BiVO4 Photoelectrocatalytic Hydrogen Evolution
Source: Molecules. 2022 Nov 12;27(22):7806. doi: 10.3390/molecules27227806 (PMC9695294; doi:10.3390/molecules27227806)
Supplement: Supplementary file 1 [file molecules-27-07806-s001.zip › molecules-2000276-SI.pdf]

Supplementary information

Effect of Functionalized Benzene Derivatives as Potential Hole Scavengers for $BiVO_4$ and rGO-$BiVO_4$ Photoelectrocatalytic Hydrogen Evolution

Tayebeh Sharifi[1], Marin Kovačić[1,*], Monika Belec[1], Klara Perović[1], Marin Popović[2], Gabrijela Radić[1], Boštjan Žener[3], Anamarija Pulitika[1], Marijana Kraljić Roković[1], Urška Lavrenčič Štangar[3], Ana Lončarić Božić[1], Hrvoje Kušić[1,*]

[1] Faculty of Chemical Engineering and Technology, University of Zagreb, Marulićev trg 19, HR-10000 Zagreb, Croatia

[2] Department of Safety and Protection, Karlovac University of Applied Sciences, Trg Josipa Juraja Strossmayera 9, HR-47000 Karlovac, Croatia

[3] Faculty of Chemistry and Chemical Technology, University of Ljubljana, Večna pot 113, SI-1000 Ljubljana, Slovenia

* Correspondence: mkovacic@fkit.hr (M.K.); hkusic@fkit.hr (H.K.); Tel.: +385-1-4597-145 (M.K.); +385-1-4597-160 (H.K.)

To whom correspondence should be addressed:
e-mails: mkovacic@fkit.hr (M.K.); hkusic@fkit.hr (H.K.)

Supplemental Materials and Methods

Thermogravimetric analysis (TGA) was performed using a Q500 thermogravimetric analyzer (TA Instruments, USA). For this purpose, ~5 mg of samples were weighed onto clean Pt pans and covered with a pierced Pt lid. Thermogravimetric analysis was performed in the temperature range of 40 °C to 800 °C at a ramp of 20 °C min^{-1} and a nitrogen furnace purge flow rate of 40 mL min^{-1}.

Figure captions

Figure S1. Isosurface plots of HOMO and LUMO frontier orbitals of PH, BA, BA^-, SA^-, 5-ASA, and 5-ASA ZW

Figure S2. Thermogravimetric analysis (TGA) of GO and rGO samples in (A) N_2 atmosphere and (B) air

Figure S3. Powder X-ray diffraction (XRD) patterns of GO and rGO

Figure S4. LSV of $BiVO_4$ and rGO-$BiVO_4$ photoelectrodes in NaCl solution (0.1 M) containing different concentrations of SFBDs; PH (A and B), BA (C and D), SA (E and F), and 5-ASA (G and H) in the dark and under illumination

Figure S5. OCP of $BiVO_4$ (A, C, E, G) and rGO-$BiVO_4$ (B, D, F, H) photoelectrodes in NaCl solution (0.1 M) with simple functionalized benzene derivatives

Figure S6. EIS of $BiVO_4$ (A, C, E, G) and rGO-$BiVO_4$ (B, D, F, H) in the dark and under light illumination in NaCl (0.1 M) with simple functionalized benzene derivatives, with inlays corresponding to response in NaCl (0.1 M) alone

Figure S1

**Compound**	**Structure**	**HOMO isosurface map**	***E*(HOMO), eV**	**LUMO isosurface map**	***E*(LUMO), eV**
phenol (PH)	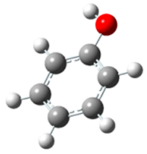	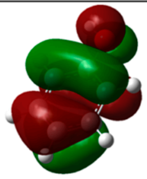	–6.422	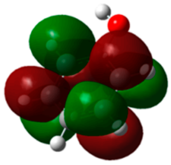	–0.490
benzoic acid (BA)	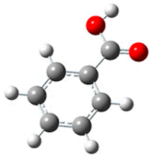	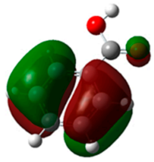	–7.483	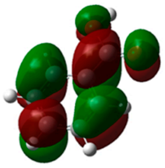	–1.714
benzoate (BA^-)	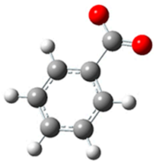	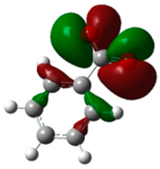	–5.742	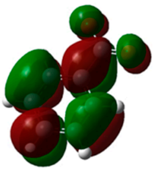	–0.544
salicylic acid (SA)	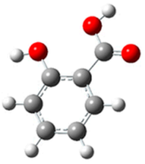	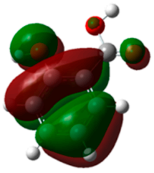	–6.721	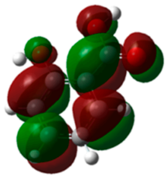	–1.605
salicylate (SA^-)	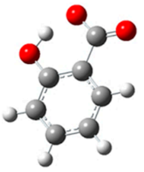	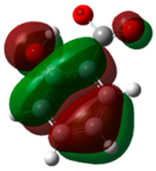	–5.850	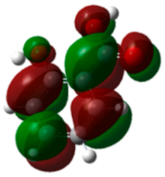	–0.626
5-aminosalicylic acid (5-ASA)	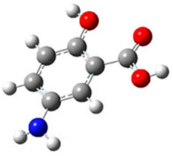	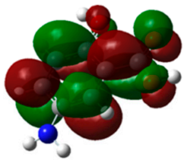	–5.660	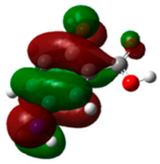	–1.551
5-aminosalicylic acid zwitterion (5-ASA ZW)	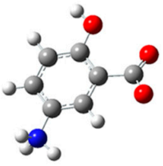	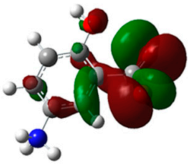	–5.850	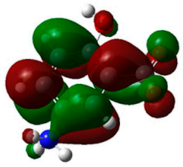	–1.061

Figure S2

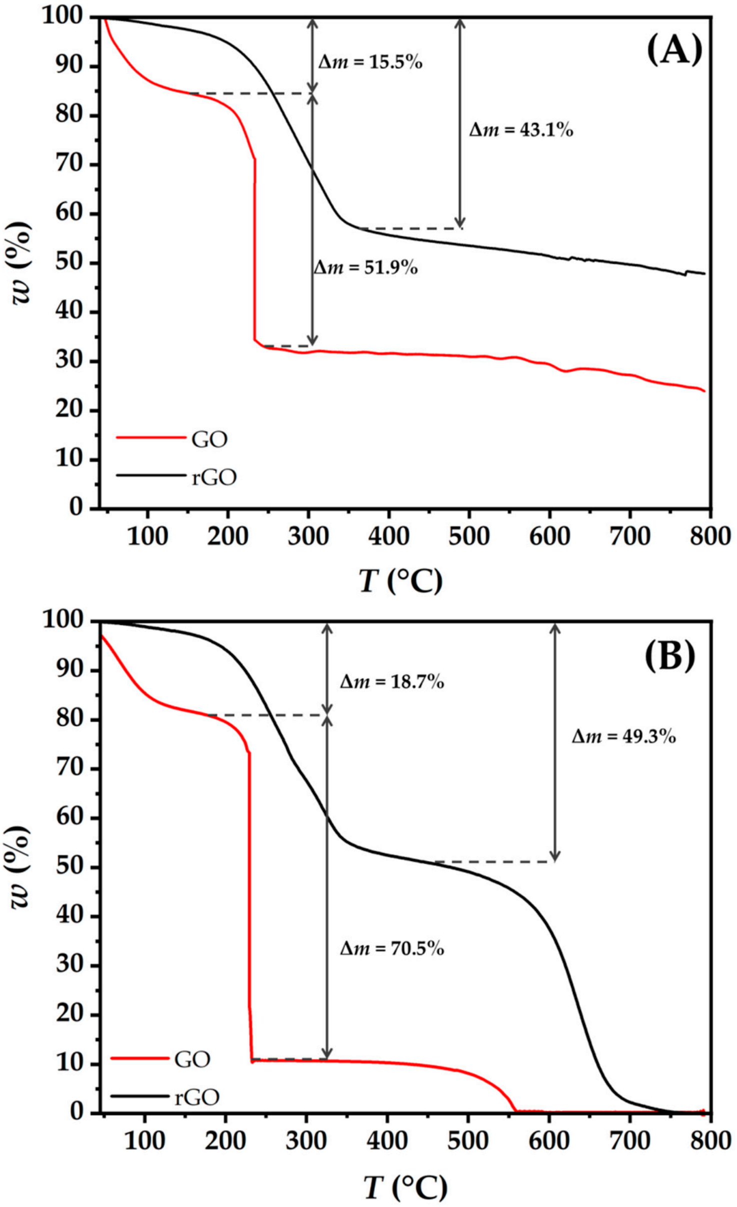

Figure S3

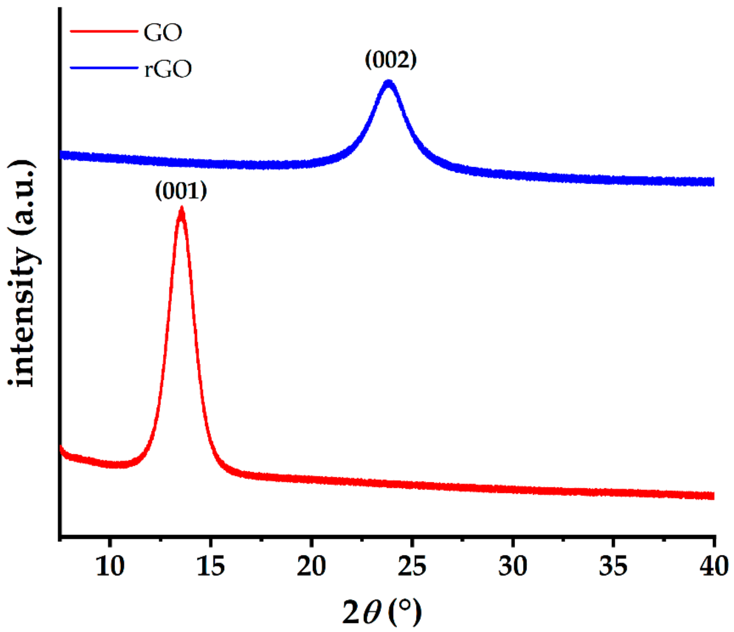

Figure S4

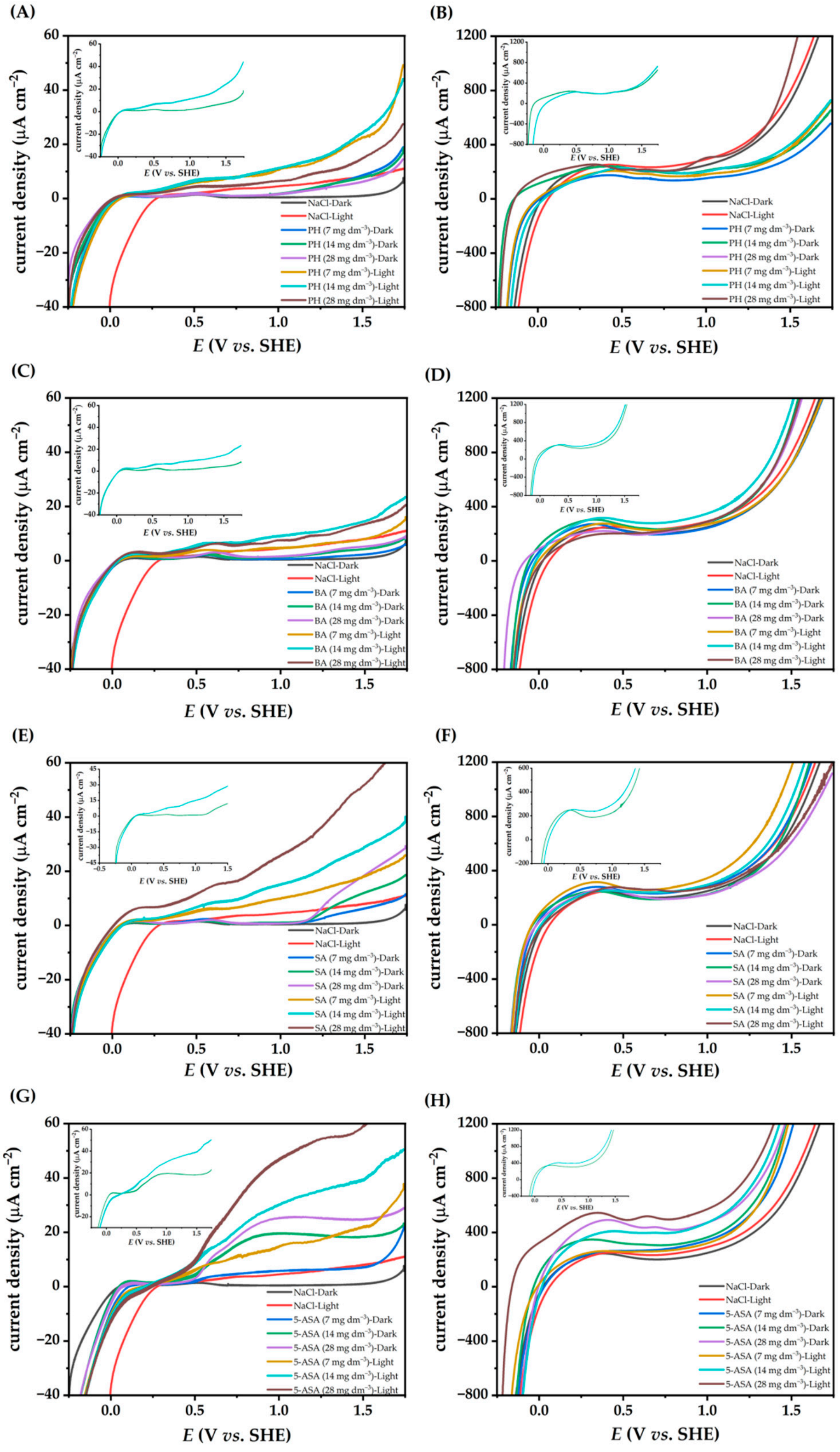

(A)
current density (μA cm⁻²)
E (V vs. SHE)
NaCl-Dark
NaCl-Light
PH (7 mg dm⁻³)-Dark
PH (14 mg dm⁻³)-Dark
PH (28 mg dm⁻³)-Dark
PH (7 mg dm⁻³)-Light
PH (14 mg dm⁻³)-Light
PH (28 mg dm⁻³)-Light
(B)
current density (μA cm⁻²)
E (V vs. SHE)
NaCl-Dark
NaCl-Light
PH (7 mg dm⁻³)-Dark
PH (14 mg dm⁻³)-Dark
PH (28 mg dm⁻³)-Dark
PH (7 mg dm⁻³)-Light
PH (14 mg dm⁻³)-Light
PH (28 mg dm⁻³)-Light
(C)
current density (μA cm⁻²)
E (V vs. SHE)
NaCl-Dark
NaCl-Light
BA (7 mg dm⁻³)-Dark
BA (14 mg dm⁻³)-Dark
BA (28 mg dm⁻³)-Dark
BA (7 mg dm⁻³)-Light
BA (14 mg dm⁻³)-Light
BA (28 mg dm⁻³)-Light
(D)
current density (μA cm⁻²)
E (V vs. SHE)
NaCl-Dark
NaCl-Light
BA (7 mg dm⁻³)-Dark
BA (14 mg dm⁻³)-Dark
BA (28 mg dm⁻³)-Dark
BA (7 mg dm⁻³)-Light
BA (14 mg dm⁻³)-Light
BA (28 mg dm⁻³)-Light
(E)
current density (μA cm⁻²)
E (V vs. SHE)
NaCl-Dark
NaCl-Light
SA (7 mg dm⁻³)-Dark
SA (14 mg dm⁻³)-Dark
SA (28 mg dm⁻³)-Dark
SA (7 mg dm⁻³)-Light
SA (14 mg dm⁻³)-Light
SA (28 mg dm⁻³)-Light
(F)
current density (μA cm⁻²)
E (V vs. SHE)
NaCl-Dark
NaCl-Light
SA (7 mg dm⁻³)-Dark
SA (14 mg dm⁻³)-Dark
SA (28 mg dm⁻³)-Dark
SA (7 mg dm⁻³)-Light
SA (14 mg dm⁻³)-Light
SA (28 mg dm⁻³)-Light
(G)
current density (μA cm⁻²)
E (V vs. SHE)
NaCl-Dark
NaCl-Light
5-ASA (7 mg dm⁻³)-Dark
5-ASA (14 mg dm⁻³)-Dark
5-ASA (28 mg dm⁻³)-Dark
5-ASA (7 mg dm⁻³)-Light
5-ASA (14 mg dm⁻³)-Light
5-ASA (28 mg dm⁻³)-Light
(H)
current density (μA cm⁻²)
E (V vs. SHE)
NaCl-Dark
NaCl-Light
5-ASA (7 mg dm⁻³)-Dark
5-ASA (14 mg dm⁻³)-Dark
5-ASA (28 mg dm⁻³)-Dark
5-ASA (7 mg dm⁻³)-Light
5-ASA (14 mg dm⁻³)-Light
5-ASA (28 mg dm⁻³)-Light

Figure S5

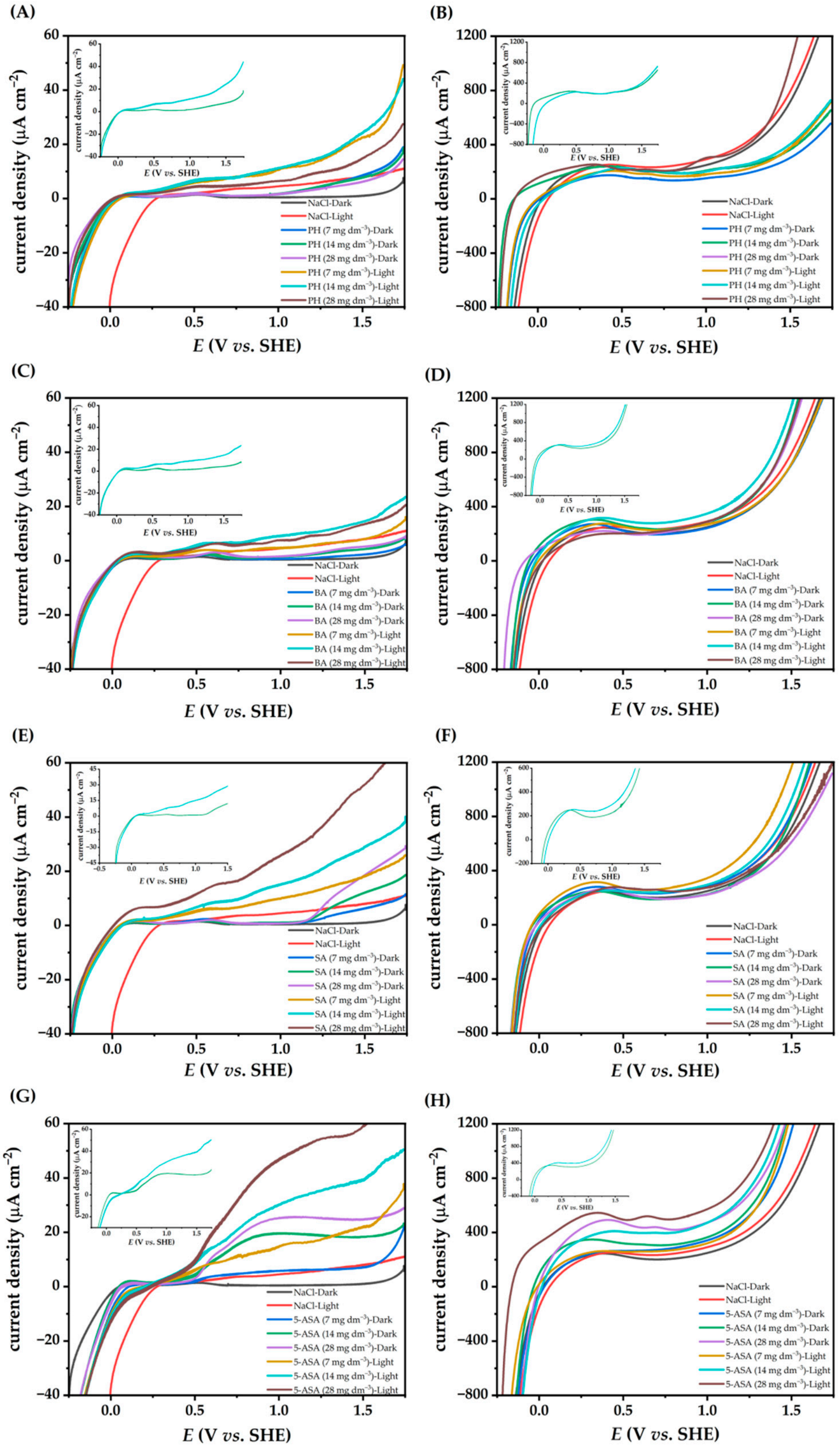
(A)
(B)
(C)
(D)
(E)
(F)
(G)
(H)
current density (μA cm^{-2})
E (V vs. SHE)
NaCl-Dark
NaCl-Light
PH (7 mg dm^{-3})-Dark
PH (14 mg dm^{-3})-Dark
PH (28 mg dm^{-3})-Dark
PH (7 mg dm^{-3})-Light
PH (14 mg dm^{-3})-Light
PH (28 mg dm^{-3})-Light
BA (7 mg dm^{-3})-Dark
BA (14 mg dm^{-3})-Dark
BA (28 mg dm^{-3})-Dark
BA (7 mg dm^{-3})-Light
BA (14 mg dm^{-3})-Light
BA (28 mg dm^{-3})-Light
SA (7 mg dm^{-3})-Dark
SA (14 mg dm^{-3})-Dark
SA (28 mg dm^{-3})-Dark
SA (7 mg dm^{-3})-Light
SA (14 mg dm^{-3})-Light
SA (28 mg dm^{-3})-Light
5-ASA (7 mg dm^{-3})-Dark
5-ASA (14 mg dm^{-3})-Dark
5-ASA (28 mg dm^{-3})-Dark
5-ASA (7 mg dm^{-3})-Light
5-ASA (14 mg dm^{-3})-Light
5-ASA (28 mg dm^{-3})-Light

Figure S6

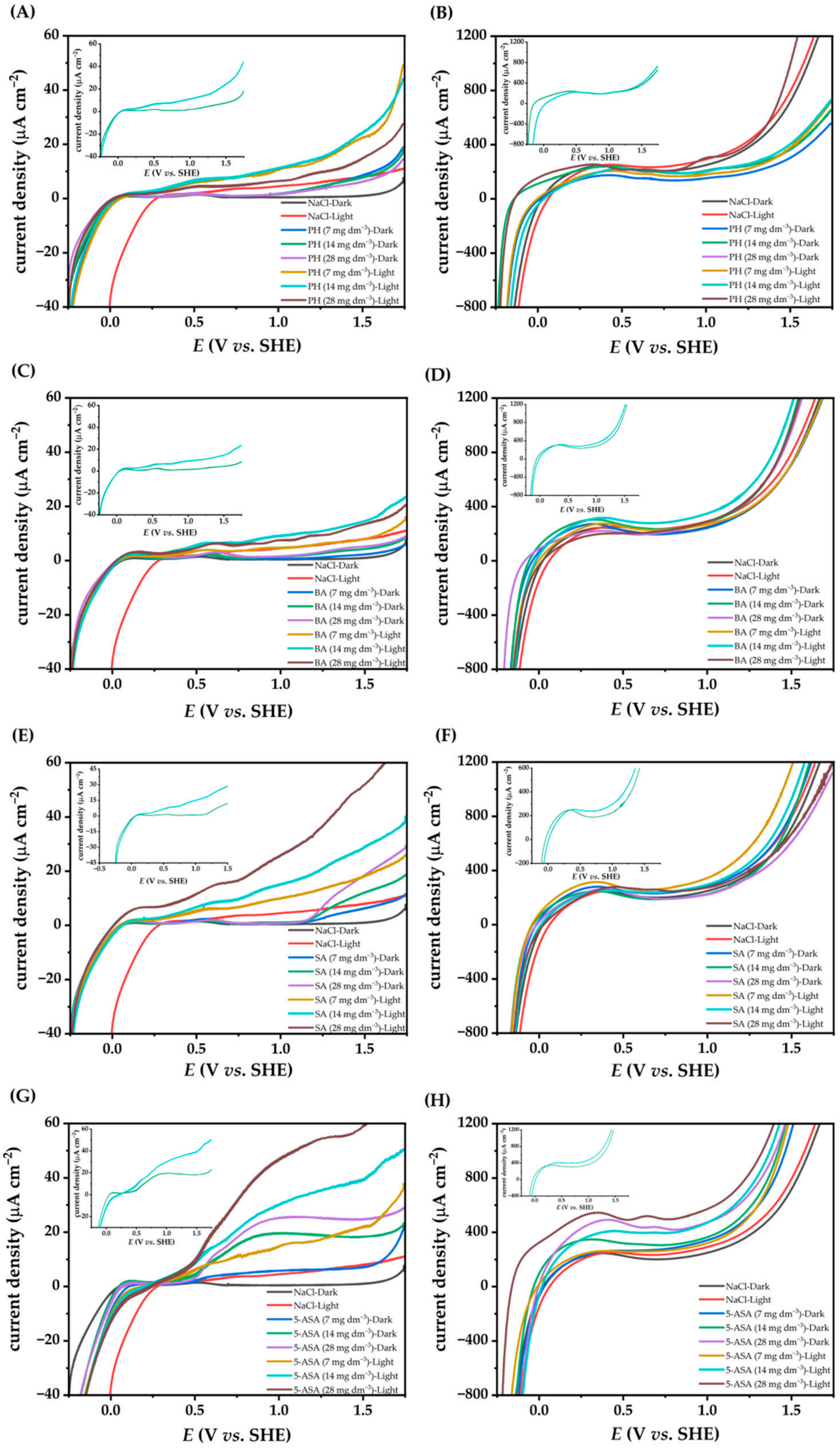
(A)
(B)
(C)
(D)
(E)
(F)
(G)
(H)
current density (μA cm−2)
E (V vs. SHE)
NaCl-Dark
NaCl-Light
PH (7 mg dm−3)-Dark
PH (14 mg dm−3)-Dark
PH (28 mg dm−3)-Dark
PH (7 mg dm−3)-Light
PH (14 mg dm−3)-Light
PH (28 mg dm−3)-Light
BA (7 mg dm−3)-Dark
BA (14 mg dm−3)-Dark
BA (28 mg dm−3)-Dark
BA (7 mg dm−3)-Light
BA (14 mg dm−3)-Light
BA (28 mg dm−3)-Light
SA (7 mg dm−3)-Dark
SA (14 mg dm−3)-Dark
SA (28 mg dm−3)-Dark
SA (7 mg dm−3)-Light
SA (14 mg dm−3)-Light
SA (28 mg dm−3)-Light
5-ASA (7 mg dm−3)-Dark
5-ASA (14 mg dm−3)-Dark
5-ASA (28 mg dm−3)-Dark
5-ASA (7 mg dm−3)-Light
5-ASA (14 mg dm−3)-Light
5-ASA (28 mg dm−3)-Light
